# Consensus on the use of oral isotretinoin in dermatology - Brazilian Society of Dermatology^☆^^☆☆^

**DOI:** 10.1016/j.abd.2020.09.001

**Published:** 2020-10-03

**Authors:** Ediléia Bagatin, Caroline Sousa Costa, Marco Alexandre Dias da Rocha, Fabíola Rosa Picosse, Cristhine Souza Leão Kamamoto, Rodrigo Pirmez, Mayra Ianhez, Hélio Amante Miot

**Affiliations:** a Department of Dermatology, Escola Paulista de Medicina, Universidade Federal de São Paulo, São Paulo, SP, Brazil; b Dermatology Discipline, Universidade Federal do Piauí, Piauí, PI, Brazil; c Private Clinic, São Paulo, SP, Brazil; d Centro de Estudos dos Cabelos, Instituto de Dermatologia Professor Rubem David Azulay, Santa Casa da Misericórdia do Rio de Janeiro, Rio de Janeiro, RJ, Brazil; e Department of Tropical Medicine and Dermatology, Universidade Federal de Goiás, Goiânia, GO, Brazil; f Department of Dermatology, Faculdade de Ciências Médicas e Biológicas de Botucatu, Universidade Estadual Paulista, Botucatu, SP, Brazil

**Keywords:** Acne vulgaris, Dermatitis, seborrheic, Isotretinoin, Rosacea, Vitamin A

## Abstract

**Background:**

Isotretinoin is a synthetic retinoid, derived from vitamin A, with multiple mechanisms of action and highly effective in the treatment of acne, despite common adverse events, manageable and dose-dependent. Dose-independent teratogenicity is the most serious. Therefore, off-label prescriptions require strict criteria.

**Objective:**

To communicate the experience and recommendation of Brazilian dermatologists on oral use of the drug in dermatology.

**Methods:**

Eight experts from five universities were appointed by the Brazilian Society of Dermatology to develop a consensus on indications for this drug. Through the adapted DELPHI methodology, relevant elements were listed and an extensive analysis of the literature was carried out. The consensus was defined with the approval of at least 70% of the experts.

**Results:**

With 100% approval from the authors, there was no doubt about the efficacy of oral isotretinoin in the treatment of acne, including as an adjunct in the correction of scars. Common and manageable common adverse events are mucocutaneous in nature. Others, such as growth retardation, abnormal healing, depression, and inflammatory bowel disease have been thoroughly investigated, and there is no evidence of a causal association; they are rare, individual, and should not contraindicate the use of the drug. Regarding unapproved indications, it may represent an option in cases of refractory rosacea, severe seborrheic dermatitis, stabilization of field cancerization with advanced photoaging and, although incipient, frontal fibrosing alopecia. For keratinization disorders, acitretin performs better. In the opinion of the authors, indications for purely esthetic purposes or oil control are not recommended, particularly for women of childbearing age.

**Conclusions:**

Approved and non-approved indications, efficacy and adverse effects of oral isotretinoin in dermatology were presented and critically evaluated.

## Introduction

Oral isotretinoin (13-cis-retinoic acid) is a retinoid, derived from vitamin A. It was synthesized in 1955, but it was only in 1973 that studies on its use in psoriasis, genetic disorders of keratinization, cystic acne, and basal cell carcinoma began. In the 1980s, it became the most effective option for treating nodular-cystic acne, and is currently indicated for moderate forms resistant to other treatments. It was approved for acne in the United States in 1982, in 1983 in Europe, and in 1990 in Brazil, revolutionizing the treatment of severe forms of acne.^1^, ^2^, ^3^, ^4^, ^5^

Clinical (mucocutaneous) and laboratory (liver function and lipid profile) side effects are dose-dependent, predictable, manageable and reversible, except for teratogenicity. Acne is the only approved indication, although many off-label uses have been reported.^6^, ^7^, ^8^

Isotretinoin acts as a prodrug, being converted into all-trans-retinoic acid (ATRA) in the cytoplasm of cells to be transported to the nucleus, where it binds to the nuclear retinoic acid receptor (RAR and RXR), isoforms a, b, and g.^9^ The known mechanisms of action are normalization of infundibular hyperkeratinization, inhibition of the production of cytokeratins 1, 10, and 14, filaggrin and matrix metalloproteinases (MMPs), and increase of cytokeratins 7, 13, and 19, laminin B1, and IL-1. Effects on proliferation, differentiation, apoptosis, and cell renewal, in addition to immunomodulation, are related to the regulation of gene expression, influencing nuclear transcription factors. There is activation of some genes (tumor suppressors or apoptotic, such as p53 and BAX and coding for collagen and fibronectin production) and inhibition of others (involved in lipid metabolism).^10^, ^11^, ^12^ On apoptosis, ATRA increases the expression of the forkhead box O3 transcription factor (FOXO3), activates the tumor necrosis factor-related apoptosis-inducing ligand (TRAIL) pathway and produces FOXO1 caspases, interrupting the cell cycle, by expressing the genes p21, 27 and 53.^13^, ^14^, ^15^, ^16^ Activation of FOXO1, a negative co-regulator of the androgen receptor, peroxisome proliferator-activated receptor-gamma (PPAR gamma), and liver X receptor-[alpha] sterol response element binding protein-1c (SREBP-1c), reduces lipogenesis. The attenuation of the mechanistic target of rapamycin complex 1 (mTORC1) stimulates the expression of PPAR gamma and SREBP-1c.^16^ Due to the negative regulation of genes related to insulin-like growth factor 1 (IGF1)/phosphatidylinositol 3-kinase (PI3K)/AKT (protein kinase B)/mTORC1 pathway and positive regulation of those responsible for FOXO1 and FOXO3/TRAIL/caspase pathways, there is suppression of sebogenesis and apoptosis of sebocytes.^16^, ^17^, ^18^ The activation of the p53 pathways represents the interconnection between the signaling pathways regulated positively or negatively by isotretinoin. The BAX protein induces apoptosis of keratinocytes with mutations induced by UV radiation; its expression is reduced by isotretinoin due to its anti-carcinogenic action.^14^ It is the only drug that alone acts on the four etiopathogenic factors of acne: it reduces acroinfundibular hyperkeratinization and comedogenesis; suppresses sebogenesis, by reducing the size and activity of sebaceous glands by up to 90%; decreases the population of *Cutibacterium acnes* (*C. acnes*), formerly called *Propionibacterium acnes* (*P. acnes*) due to changes in the follicular microenvironment; and modulates inflammation by the negative regulation of toll-like 2 and 4 membrane receptors (TLR-2 and 4) in keratinocytes, sebocytes, monocytes, corneal cells, and immune cells.^18^, ^19^, ^20^, ^21^, ^22^ These receptors are activated by identification of the molecular patterns of *C. acnes* and, when inhibited, there is downregulation of the nuclear factor kappa B (NF-κB) pathways, which triggers the production of cytokines (IL-8, IL-1 β, IL-17, IFNγ) and activator protein 1 (AP-1) responsible for the synthesis of MMPs.^13^, ^20^ After 40 years, it is believed that not all mechanisms of action on the skin and other organs are elucidated. However, existing knowledge explains the efficacy in acne vulgaris and adverse events, justifying unapproved, off-label indications.^23^, ^24^, ^25^

The actions of isotretinoin in different cells, explaining its beneficial effects and adverse events, are summarized in Table 1.

**Table 1:** Mechanism of action of oral isotretinoin in different cell types

Type of cell	Isotretinoin effect (desired or adverse)
Sebocyte	↓ Sebum production; acne improvement
Neural crest cells	Teratogenicity
Hippocampus cells	Reduction of hippocampal neurogenesis: depression
Keratinocyte	Mucocutaneous adverse effects – cornification alteration
Hair follicle cells	Telogen effluvium
Miotic	CPK release
Hepatocyte	Release of transaminases
Intestinal epithelium	Inflammatory bowel disease
Meibomian cells	Dry eyes

The purpose of this article is to present a consensus on the effects of oral isotretinoin on the skin and its indications for acne and others conditions not yet approved, despite the existence of relevant data in the consulted literature.

## Methods

Eight dermatologists, experts in isotretinoin, were nominated to reach a consensus on the use of oral isotretinoin in dermatology, following the adapted DELPHI methodology. In the first phase, relevant topics were discussed and the text was structured, each author responsible for a different topic; in the second phase, a bibliographic review and drafting of the texts was carried out. The databases consulted were as follows: Cochrane Skin Group Specialized Register, Cochrane Library, MEDLINE, PubMed, Embase, and LILACS. The literature in Portuguese, English, and Spanish was searched using the following keywords isotretinoína, aliança terapêutica, dermatologia, acne vulgar, dermatite seborreica, rosácea, psoríase, ceratose actínica, envelhecimento da pele, dermatology, oral isotretinoin, off label use, off label prescribing, acne, skin diseases, rosacea, photoaging of skin, actinic keratosis, seborrheic dermatitis, psoriasis, alopecia. The first author and the mentor were responsible for compiling a single text and sending it for review by the others. In the third phase, both authors assessed the consensus on the texts; what reached 70% consensus remained in the final version.

## Results/Discussion

Two topics on oral isotretinoin in dermatology were defined: acne vulgaris and relevant, off-label indications (inflammatory diseases of the skin and scalp, photoaging, and field cancerization). The results and discussion of each topic are presented below.

### Acne vulgaris

Isotretinoin is the only drug that acts on all etiopathogenic factors of acne vulgaris, remaining the only monotherapy capable of providing prolonged remission or cure in up to 80% of patients, with one treatment cycle.

Acne vulgaris, a chronic, immune-mediated, multifactorial inflammatory disease that affects the pilosebaceous unit is among the three most prevalent dermatoses worldwide.^26^ It can generate physical (scarring) and psychological sequelae, second only to eczema.^27^ It affects 80% to 90% of the world population at some stage in life, with a peak prevalence between 16–20 years.^28^, ^29^, ^30^, ^31^, ^32^ According to a survey among members of the Brazilian Society of Dermatology and other epidemiological studies, acne vulgaris is the leading cause of dermatology consultations.^33^, ^34^, ^35^

**Figure 1:**
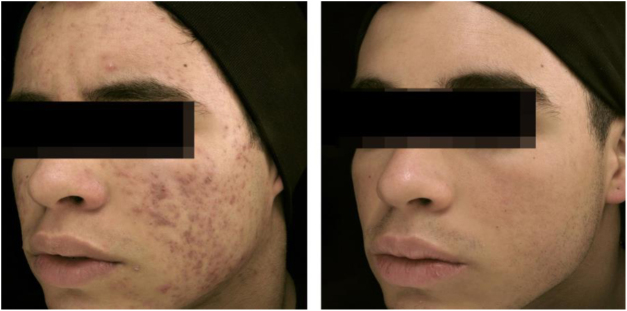
18-year-old teenager, with moderate inflammatory acne on the face and trunk for four years, presenting scars, with a relevant negative impact on quality of life. The patient had been submitted to four cycles of oral cyclin, associated with topical combination of benzoyl peroxide and adapalene, with improvement and recurrence after two to three months. During the last cycle, the clinical picture worsened. The patient was treated with oral isotretinoin, 40 mg/kg/day (0.6 mg/kg/day), with total lesion regression after four months and maintenance for another month (total dose = 100 mg/kg/day) – regimen based on recent publications.^77^, ^80^, ^81^, ^82^ Photos before and after treatment with oral isotretinoin. Maintenance treatment with adapalene 0.1% gel, for 12 months. There was no recurrence.

**Figure 2:**
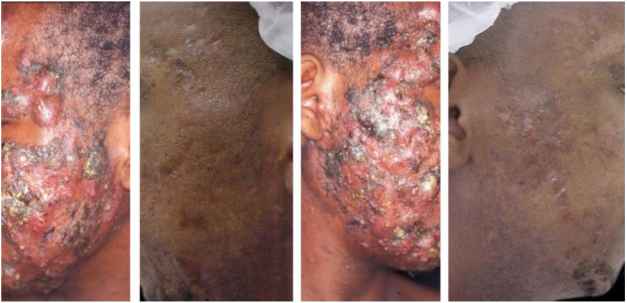
A 22-year-old patient with acne conglobata on the face alone for 15 months. Previously treated with oral antibiotics and topical products (whose names the patient was unable to report), without improvement. Treatment with isotretinoin 20 mg/day (0.3 mg/kg/day) and prednisone 40, 30, 20, and 10 mg/day every seven days was initiated. The duration of treatment, always with the same daily dose, was 18 months (160 mg/kg), until complete resolution of the lesions. A maintenance treatment with benzoyl peroxide 5% was maintained for 12 months. There was no recurrence.

The clinical effectiveness of oral isotretinoin is superior to other acne treatments, promoting healing or prolonged remission,^36^ improving quality of life, and reducing psychosocial damage; however, adverse effects are observed in up to 90% of patients.^36^, ^37^, ^38^, ^39^ Figure 1, Figure 2 illustrate patients treated with oral isotretinoin, with healing of acne and absence of recurrence after two years of follow-up. Some controversies about rare and serious events, particularly depression, suicide, and inflammatory bowel disease (IBD), have not been proven to be causally associated.^40^ It was approved for severe acne (conglobata and nodular-cystic), but evidence demonstrated in controlled randomized clinical trials (CRCTs) since 1980, in systematic reviews (SRs), consensuses, and recommendations of dermatology societies allowed to expand the indication for nodular-cystic and moderate papulopustular forms resistant to other treatments, with tendency to scarring, and emotional and social functional impairment.^1^, ^2^, ^36^, ^38^, ^40^, ^41^, ^42^, ^43^, ^44^, ^45^, ^46^, ^47^, ^48^, ^49^, ^50^, ^51^, ^52^, ^53^, ^54^, ^55^, ^56^, ^57^, ^58^, ^59^, ^60^, ^61^, ^62^, ^63^, ^64^, ^65^, ^66^, ^67^, ^68^, ^69^, ^70^, ^71^, ^72^, ^73^, ^74^ A single course of the drug leads to cure in two-thirds of patients. Recurrences may take place, but they are milder and manageable with topical treatments.^66^ Some characteristics of the disease favor recurrence and retreatment.^66^, ^75^, ^76^, ^77^, ^78^

Table 2 presents indications, contraindications, and warnings for use, in addition to the acne characteristics related to the need for retreatment. Despite 31 CRCTs, two SRs concluded that studies with better methodology and less heterogeneous as to the efficacy outcomes are needed, particularly comparative studies between the use of oral isotretinoin and the use of oral antibiotics associated to topical agents (combinations of retinoid and benzoyl peroxide). In addition, studies should include a greater number of participants, mainly females, prepubescents, and patients with trunk involvement; the long-term efficacy outcomes must be assessed, especially the superiority of this drug in terms of cure or prolonged remission of acne.^40^, ^70^

**Table 2:** Indications, contraindications, warnings, and characteristics of isotretinoin in the treatment of acne, and conditions related to the need for retreatment

Indications for use	Conditions related to the need for more than one course of treatment	Contraindications for use	Warnings
Severe acne (conglobata and nodular-cystic)	Age ˂ 16 years and family history of severe acne	Concomitant treatment with antibiotics from the tetracycline group (risk of intracranial hypertension)	Oral isotretinoin may reduce serum levels of carbamazepine and phenytoin (caution when using concomitantly)

Moderate acne (nodular-cystic or papular-pustular) with resistance to initial treatments, tendency to scarring, significant emotional impairment or impaired social functions	Long-lasting acne	Liver failure	Progesterone microdoses (“mini-pills”) are unsuitable for necessary contraception
	Female sex and hormonal changes (polycystic ovary syndrome)	Pregnancy and breastfeeding	Do not donate blood during treatment and up to 30 days after termination (risk of accidental exposure of pregnant women to the drug)
	Drug intake outside mealtimes (except isotretinoin-lidose, not available in Brazil)	Pre-existing hypervitaminosis A	Avoid sun exposure
	Presence of macrocomedones	Severe dyslipidemia	
	Treatment interruption before total resolution	Allergic to soy and parabens (excipient and preservative in capsule formulation, respectively)	
	“Hormonal athletes”: patients with hyperandrogenism secondary to the use of androgen-containing drugs		

The approved dose in the package insert is 0.5 to 1 mg/kg/day, taken after meals, due to the lipophilic character of the molecule, except for the isotretinoin-lidose variant, not available in Brazil, which can be administered while fasting.^66^, ^79^ A CRCT compared the daily dose at two meals *vs*. single dose; no difference was observed in efficacy, but adverse effects were more frequent with the use of a single dose.^67^ However, there is a preference for a single dose, due to greater treatment adherence.^79^ An SR analyzed CRCT with different daily doses and therapeutic regimens.^40^ The effectiveness was greater among groups that received a conventional or low dose (<0.5 mg/kg/day) daily when compared with intermittent use, in monthly pulses or alternate days.^56^, ^62^, ^68^ Mild adverse effects were more frequently observed with daily and continuous use, under low or conventional doses.^55^, ^56^, ^60^, ^62^, ^68^ Intermittent use was less effective and is not recommended.^36^ Recently, studies have shown a tendency toward lower daily doses (0.1–0.5 mg/kg, up to 5 mg) for moderate acne, with a longer duration, up to 18 months, presenting less adverse events, better tolerability, and recurrence rates similar to those observed with conventional dose, maintaining treatment for two to four months after total lesion resolution.^38^, ^71^, ^80^, ^81^, ^82^ Prolonged duration is necessary in severe cases and in extra-facial involvement.^77^ The approved total dose (120 to 150 mg/kg) is maintained in clinical studies, consensuses, and dermatological practice; however, there has never been a CRCT-based rationale.^81^, ^82^, ^83^, ^84^ Studies with a better methodology have shown that a fixed total dose is not the best reference for the duration of treatment, which must consider individual conditions, regression of the disease, and maintenance for two to four months after total resolution of the lesions.^40^, ^71^, ^77^, ^81^, ^82^, ^84^, ^85^

Dose-dependent mucocutaneous clinical adverse events are common, such as cheilitis, which affects 90–100% of patients, and cutaneous, ocular, and nasal mucosa xerosis. They are manageable with the use of lip lubricants, and ocular and nasal emollients, and they regress with dose reduction or treatment suspension.^36^ Other rare events include alopecia, pyogenic granuloma, photosensitivity, arthralgia, myalgia, headache, anorexia, insomnia, and irritability.^70^ The most serious risk is teratogenicity, which is dose-independent.^4^, ^40^ Pregnancy can have a normal course in 65–85% of cases, but there is a risk of miscarriage (10.9–20%) and embryopathies (18–28%), with craniofacial, central nervous system, thymus, and cardiovascular anomalies.^4^, ^5^, ^6^, ^7^ The possibility of pregnancy must be ruled out (by testing and waiting for menstruation); prescription of oral contraceptives or intrauterine devices associated with condoms is mandatory for women of childbearing age, unless hysterectomized.^4^, ^83^ The measurement of blood chorionic beta-gonadotropin should be requested beforehand and monthly, during treatment. There are no risks for future pregnancies, which are authorized one month after the end of treatment.^83^, ^86^

The hypothesis of triggering psychiatric disorders and IBD has caused numerous lawsuits in the United States. However, no CRCT has demonstrated these associations.^40^, ^70^ Qualitative analysis of 14 non-randomized studies on serious adverse events, nine on psychiatric adverse events, and seven on IBD did not demonstrate an increased risk.^40^, ^87^, ^88^, ^89^, ^90^, ^91^, ^92^, ^93^, ^94^, ^95^ Two other SR with meta-analysis assessed depression and IBD, and did not detect an increased risk due to exposure to isotretinoin. In contrast, reduced levels of depression have been demonstrated in comparison with topical therapy.^96^

Acne is related to psychosocial damage, increased risk of depression, and suicide, conditions already present in adolescence.^81^, ^97^ Some subgroups may be more susceptible to depression and psychosis induced by isotretinoin in an idiosyncratic manner.^40^, ^98^ Personal and/or family history of depression are not contraindications to the use of the drug in low daily doses and monitoring of mood and behavior in the daily routine, with the help of a psychiatrist.^81^

Increased risk of IBD has already been associated with previous use of antibiotics and acne itself.^72^, ^91^, ^99^ Thus, a history of IBD is not a contraindication for isotretinoin.

Acne flares in the first eight weeks of treatment are related to sebocyte apoptosis, antigen release, and intense inflammatory response, being observed in 15–18% of patients, with spontaneous resolution.^100^ However, they can mimic fulminant acne, without systemic symptoms, and with intense inflammation, ulceration, scabs, and scars.^101^ The drug should be kept at a low dose and associated with prednisone, 0.5–1 mg/kg/day for two to four weeks or until resolution. Severe and extensive acne (face, chest, and back), macrocomedones, and family history indicate initiation of treatment with low daily dose (0.1–0.2 mg/kg), associated with prednisone in the first two to four weeks; low dose is maintained for eight weeks and may or may not be increased gradually, along with fractional corticosteroid withdrawal.^36^, ^42^, ^100^, ^101^, ^102^

Laboratory alterations correspond to 2% of the detected adverse events.^70^ The serum dosages most frequently altered, according to SRs and meta-analyses, are as follows: triglycerides (44%), total cholesterol, LDL-cholesterol (33%), and liver enzymes (11%).^70^, ^103^ There is no evidence that these elevations increase cardiovascular risk.^36^, ^40^ Previous liver and lipid profiles are recommended, repeated after one month and every three months.^83^ Analysis of laboratory monitoring concluded that tests requested less frequently are safe and economical, since changes are rare or discreet and reversible. Thus, a lipid and hepatic profile is recommended at baseline and after two months; subsequently, only the altered exams should be repeated, according to the patient's medical history.^102^ Thus, a lipid and hepatic profile is recommended at baseline and after two months; subsequently, only the altered exams should be repeated, according to the patient's medical history.^102^ However, some authors and even the Brazilian Unified Health System (Sistema Único de Saúde [SUS]) still recommend frequent monitoring. A very recent study also concluded that the quality of care for patients with acne can be improved by reducing the frequency of assessment of lipids and hepatic function and eliminating the blood count assessment.^103^ The possibility of interference with strength, fatigue, and muscle endurance was investigated and no difference was observed in a study that compared patients with individuals who did not use isotretinoin.^104^ Thus, CPK measurements are only indicated if the patient has severe muscle pain.^105^

The risk of abnormal scarring with the use of isotretinoin was assessed; five recently published guidelines concluded that there was no evidence to delay superficial cosmetic procedures, biopsies, and dermatological surgeries without involvement of muscle planes (Table 3). A retrospective observational study demonstrated no tendency to hypertrophic scarring and keloid among acne patients who used oral isotretinoin.^106^ On the contrary, some recent publications have emphasized that the use of lasers is safe, even producing better results in the case of scars, if started in the last month of treatment with isotretinoin.^107^, ^108^, ^109^, ^110^, ^111^

**Table 3:** Dermatological procedures for patients currently using or recently having used oral isotretinoin

Safe	Not recommended
Manual dermabrasion	Mechanical dermabrasion
Microdermabrasion	Deep peels
Microneedling	Ablative lasers
Ablative and non-ablative fractional lasers	Deep dermatological excisions, muscle flap
Medium and superficial peels	
Q-switched lasers and vascular lasers	
Micro-needled fractional radiofrequency	
Biopsies, superficial excisions	

### Off-label prescriptions

#### Inflammatory diseases

##### Rosacea

It is believed that isotretinoin may act on rosacea by modulating innate immunity and reducing the inflammatory response through the negative regulation of TLR-2 expression in keratinocytes. Off-label use is indicated for moderate to severe papule-pustular rosacea, at a low daily dose (0.25–0.3 mg/kg), for four months, with a slow and progressive reduction. Maintenance treatment is mandatory, with topic medication (metronidazole, azelaic acid, or ivermectin), or isotretinoin in microdoses (20 mg/week), with laboratory control and assessment of pregnancy risk.^112^, ^113^, ^114^, ^115^, ^116^, ^117^, ^118^, ^119^, ^120^, ^121^, ^122^, ^123^, ^124^, ^125^, ^126^

The use of oral isotretinoin for severe rosacea was first reported in 1981 in a German study that demonstrated efficacy and longer periods of remission when compared with usual treatments. Daily doses of 0.05 mg/kg, 0.5 mg/kg, or 1 mg/kg were used for 12–28 weeks. There was a 50% regression of inflammatory lesions in two weeks and 95% in eight weeks. Only telangiectasias and chronic conjunctivitis showed little improvement. Remissions were observed for more than 12 months. Side effects were mild cheilitis and a slight increase in triglycerides and cholesterol.^112^ A multicenter study, including 92 patients, lasting 20 weeks and using the same doses, concluded that isotretinoin is effective in rosacea refractory to previous recommended treatments.^113^ A CRCT compared isotretinoin, at a dose of 10 mg/day, with 0.025% tretinoin cream or both, for 16 weeks and another 16 weeks of maintenance with tretinoin or placebo cream in severe rosacea, with no differences and no advantage of the association. Adverse events were minimal and well tolerated.^114^

The use of oral isotretinoin in the treatment of rosacea has been reported since the 1980s, in most cases by European and American authors. It is worth mentioning the first publication in Latin America in 1994, by a Chilean author who observed, in a series of six cases treated for three to six months with a dose of 0.5 mg/kg/day, rapid remission of papules and pustules, improvement in ocular manifestations, few side effects, and maintenance of results for approximately 15 months.^115^

A multicenter, double-blinded, randomized study included 573 patients with papule-pustular and phymatous rosacea comparing different doses (0.3; 0.5; 1 mg/kg/day) *vs.* doxycycline 100 mg/day, 14 days and then 50 mg/day *vs*. placebo. After 12 weeks, the dose of 0.3 mg/kg/day was more effective than placebo, with efficacy equal to or greater than doxycycline (reduction of 90% *vs*. 83% of lesions) and fewer side effects.^116^

Another multicenter, randomized study, including 156 patients, compared the dose of 0.25 mg/kg/day (*n* = 108) *vs*. placebo (*n* = 48), for four months. The primary outcome (90% reduction in the number of lesions) was observed in 57% *vs*. 10% of the patients. Four-month recurrence was observed in 58% of patients. Studies have been suggested to investigate the minimum dose to maintain remission.^117^

To control recurrences, continuous microdoses have been proposed. Twelve patients with recurrent rosacea were treated with 10–20 mg/day for four to six months and subsequently received a maintenance dose of 0.03–0.17 mg/kg/day (mean: 0.07 mg/kg/day) for up to 33 months. There was an improvement in quality of life, suggesting that microdosing would be a better option than multiple cycles of antibiotic therapy.^118^ In another study, 25 patients were treated with a dose of 20 mg/day for four months, with rapid reduction of erythema and inflammatory lesions; subsequently, a slow dose reduction was performed for six months, up to 20 mg/week. At 11 months, 45% of cases presented recurrence.^119^

Fulminant rosacea is a unique, rare, highly inflammatory form in the center of the face, with an abrupt onset and the presence of papules, pustules, nodules, and sinus tracts draining sero-purulent, coalescent secretion. The treatment of choice is isotretinoin associated with prednisone, 40–60 mg/day. The initial daily dose of 0.2–0.5 mg/kg is recommended, increasing to 0.5–1 mg/kg for three to four months.^120^

There are no controlled and randomized studies on the use of oral isotretinoin in phymatous rosacea. A Singapore author reported, in a letter, a reduction in rhinophyma in one patient, after six months of treatment with isotretinoin, 20 mg/day, with a tendency to recurrence after eight months. That author remarked that it is an option to reduce the lesion for later surgical or laser procedures.^121^ In the last published SR, it was not possible to include studies on phyma.^122^ By suppressing the sebaceous gland and decreasing sebogenesis, isotretinoin could delay the progression of the phyma when used in the pre-fibrotic phase, with better results in young patients, but recurrence is observed after drug discontinuation.^122^, ^123^ The global consensus panel, ROSaceaCOnsensus (ROSCO), indicates isotretinoin as a therapeutic option in the severe inflammatory (papulopustular) form and in inflamed phyma, in an early stage, with a high degree of recommendation.^124^

Regarding ocular rosacea, a review article indicated the benefit and safety of isotretinoin.^125^ A recent comparative study with doxycycline, published by Brazilian ophthalmologists and dermatologists, showed that although doxycycline was more effective, isotretinoin, at a dose of 10 mg/day, also improved blepharitis and conjunctivitis, without adverse events.^126^

An SR, using the Cochrane methodology, concluded that isotretinoin has a high degree of recommendation for moderate to severe papular-pustular rosacea, relapsing cases or those unresponsive to antibiotic therapy, and for inflamed phymas. The dose of 0.25 mg/kg/day for 12–16 weeks is greater than that of doxycycline, 50–100 mg/day. Topical maintenance is always recommended.^122^

The Ibero-Latin American Rosacea Studies Group has published a treatment algorithm including low daily dose isotretinoin for the papule-pustular and hyperplastic/phymatous gland subtypes.^127^ A Canadian guideline presented the same recommendation.^128^ A review article highlights the excellent results of this drug for rosacea and recommends that dermatologists consider this option, since its safety has been determined after more than 30 years of use, reducing the use of oral antibiotics for chronic disease.^129^, ^130^ The American Society of Acne and Rosacea, in its consensus, suggests isotretinoin for diffuse mid-facial erythema with papules and pustules, granulomatous rosacea, and early phyma.^131^ A low dose is effective, with fewer side effects and good adherence. There is a need for clinical and laboratory control and attention to teratogenicity.^132^ As it affects the face, rosacea has a negative impact on quality of life and its control provides benefits in patients’ emotional, social, and professional lives.^133^

##### Seborrheic dermatitis (SD)

SD is a chronic, recurrent inflammatory dermatosis, located in areas of high concentration of sebaceous glands: face (88%), retroauricular region, scalp (70%), anterior chest (27%), lower limbs (2%), upper limbs (1%), and flexures (5%).^133^

Despite little knowledge about its etiopathogenesis, it is admitted that the efficacy of isotretinoin in SD is explained by the sebo-suppressive action and modulation of innate immunity and inflammatory response, *i.e*., downregulation of TLR-2 and the NF-κB pathway, with reduction in cytokine production. In SD, TLR-2 is activated by lipophilic fungi of the genus *Malassezia*, in adults and *Candida* spp. in infants, present in the normal skin microbiota,^134^, ^135^ explaining the option of topical treatment of SD with antifungals.^136^ Topical immunomodulators and corticosteroids are also used^137^; in extensive conditions resistant to topical treatment, systemic treatment with corticosteroids or isotretinoin may be necessary. This drug is a second-line treatment, used in clinical practice, but there is no definition of dose and duration of treatment. The need for laboratory control and pregnancy prevention is emphasized.^138^

The first report of successful use of isotretinoin in SD, in a low daily dose, was published in Germany in 2003.^139^ Subsequently, a 14-year-old adolescent with pityriasis versicolor (PV) on the back and severe acne was treated with 40 mg of isotretinoin, twice daily (1 mg/kg/day) for five months. Clinical and mycological cure of PV was observed, suggesting a role against *Malassezia* directly or by reducing the skin's lipid content due to the xerosis caused by the drug, interfering with the microbiota's conditions, since this fungus is lipophilic.^140^

A patient with severe facial SD for 22 years was treated with isotretinoin, 0.3 mg/kg/day, with improvement after 30 days; the dose was reduced to 0.15 mg/kg, every other day for two months, with complete remission.^141^ In 2017, a review of 46 cases, 40 associated with acne, 57% women, mean age 26 years, with non-responsive SD, treated with doses of 0.05–0.51 mg/kg/day (mean: 33 weeks), associated with topical ketoconazole and hydrocortisone, showed total regression or excellent response in 89% of the cases; one patient presented no improvement.^142^ A study that compared 10 mg/day, on alternate days, with salicylic acid and piroctone olamine topical treatment (shampoo and soap) for six months, in parallel groups, observed a reduction in the clinical score in both groups; however, this reduction was greater in the isotretinoin group, with reduction in the rate of sebaceous secretion and no effect on the quantity and species of *Malassezia*.^143^, ^144^ The role of *Malassezia* in the pathogenesis of SD remains controversial.

##### Psoriasis

Isotretinoin, as well as etretinate and acitretin, act in the control of psoriasis by converting keratinocytes in the cytoplasm into all-trans retinoic acid, which penetrates the nucleus, binds to nuclear receptors, and activates specific regions of DNA, involved in regulating growth and cell differentiation and apoptosis. Thus, it reduces the hyperproliferation of keratinocytes, which is one of the events involved in the pathogenesis of psoriasis.^24^

The report of four cases of extensive psoriasis in women treated with 0.6 mg/kg/day of isotretinoin associated with phototherapy, with oral 8-methoxypsoralen and exposure to UVA (PUVA), showed reduction in the number of PUVA sessions.^145^ Two randomized clinical studies described the benefit of this drug, at a dose of 0.5 mg/kg/day, associated with narrowband ultraviolet B (NB-UVB) or PUVA, for disseminated plaque psoriasis, reducing the number of phototherapy sessions. The option for isotretinoin is due to the shorter period of contraception, due to its shorter half-life in relation to etretinate or acitretin.^146^, ^147^ For the same reason, isotretinoin was used, with excellent results, in a female teenager with generalized pustular psoriasis at a dose of 1.0 mg/kg/day and in two other adult patients at doses of 1.5–2.0 mg/kg/day, for four months.^148^, ^149^ In a recent SR on the treatment of palmoplantar pustulosis, it was not possible to demonstrate evidence for any treatment, except for potent or systemic topical corticosteroids.^150^

Other systemic treatment options for psoriasis are available, such as methotrexate, cyclosporine, and a large number of immunobiologicals. Retinoid monotherapy has limited efficacy, but can be useful when combined with corticosteroids in pustular psoriasis and phototherapy in HIV-positive individuals, as it has no immunosuppressive effect.^151^

### Hidradenitis suppurativa (HS)

Isotretinoin in HS is not the treatment of choice; effectiveness is variable and can be explained by anti-inflammatory actions (TLR-2 modulation), and reduced expression of genes related to keratinocyte hyperproliferation.^24^

HS is a chronic inflammatory disease, difficult to treat, with a negative impact on quality of life, with nodules, fistulas, abscesses, and scars. Deep excision of the lesions is the curative treatment. The use of isotretinoin, alone or in association with other treatments, has been mentioned in the literature, with variable results, in the most severe forms, as an option to reduce lesions and facilitate surgery later.^152^ In a retrospective study including 209 patients, 39 treated with isotretinoin, at a dose of 0.5–1.2 mg/kg/day, for four to 12 months, 14 (36%) patients presented improvement, with benefit for performing surgery.^153^ Another recent study assessed drug combinations for HS in 31 patients and demonstrated the benefit of isotretinoin associated with spironolactone, in milder, initial cases, an ideal time to introduce treatment and prevent disease progression.^154^

#### Photoaging

Oral isotretinoin can improve the clinical, histological, and molecular characteristics of photodamage in the skin, possibly due to its conversion to all-trans retinoic acid or tretinoin.^24^ Topical use of tretinoin is the treatment of choice, with the highest level of evidence for moderate to severe photoaging.^155^, ^156^, ^157^ Its mechanisms of action are as follows: reversal of mutations in the p53 gene, reduced MMPs, increased tissue inhibition of metalloproteinase (TIMPs), and reduced loss and accelerated recovery of nuclear retinoid receptors after exposure to UV radiation.^24^, ^158^

Regarding its use in photoaging, an author from El Salvador reported, for the first time, his experience on the use of isotretinoin as an adjunct to cosmetic procedures. Despite being randomized, the study was open and uncontrolled, including 120 patients. The dose was 10 or 20 mg/day, without reference to the criterion used, three times a week, for only two months, in a group of patients undergoing varied procedures (chemical peels, botulinum toxin, collagen filling, blepharoplasty, liposuction, fat graft, facelift), without explaining whether the use was previous or concomitant. The clinical outcomes, which are difficult to assess, were as follows: pore size, pigmentation, wrinkles, thickness, elasticity, and skin color. The results were compared to those of the group that did not receive the drug and were considered better with the association.^159^ Another five studies were published by Brazilian authors (details in Table 4).^160^, ^161^, ^162^, ^163^, ^164^ One of them, which included 188 patients, only compared the clinical and histological effects of doses of 10 or 20 mg, on alternate days for two to six months, and found no differences.^161^ The two randomized studies used isotretinoin, at a dose of 20 mg, on alternate days, for three and six months, and were compared to the use of only photoprotector and moisturizer or topical tretinoin, respectively. In both cases, there was no superiority of isotretinoin in terms of clinical, histological, and immunohistochemical outcomes, except for the expression of the epidermal p53 protein, which had a significant reduction with the use of the evaluated oral drug. As for safety, no adverse clinical or laboratory events were observed, except for mild cheilitis and xerosis.^162^, ^163^

**Table 4:** Details of the six studies on oral isotretinoin for skin aging

Author, year	*n*/dose/treatment time	Outcomes
Hernandez, 2000^159^	Group 1 (*n* = 60): 10–20 mg 3 × /week	Clinical: improvement of wrinkles, skin thickness and color, pores, elasticity, and pigmented lesions
	Group 2 (*n* = 60): placebo	
	Cosmetic procedures 2 groups, 2 months	

Kalil, 2008^160^	Single group (*n* = 50): 20 mg 3 × /week, 3 months	Clinical: improvement in the general appearance of the skin, wrinkles, color, and texture
		Histological: improvement of collagen and elastotic fibers of solar elastosis

Rabello-Fonseca, 2008^161^	Group 1 (*n* = 15): 10 mg 3 × /week	Clinical: improvement in the general appearance of the skin, wrinkles, color, and texture
	Group 2 (*n* = 15): 20 mg 3 × /week	Histological: improvement of collagen and elastotic fibers of solar elastosis
	3 months	No difference between doses

Bagatin, 2010^162^	Group 1 (*n* = 16): 20 mg 3 × /week + photoprotector	Clinical: clinical improvement, profilometry, corneometry, and viscoelastic measures
	Group 2 (*n* = 16): photoprotector	
	3 months	Histological and immunohistochemical: slight improvement, no significant reduction in p53 protein expression

Bagatin, 2014^163^	Group 1 (*n* = 12): 20 mg 3 × /week + photoprotector	Clinical: improvement in patient opinion, blinded photographic assessment, quality of life
	Group 2 (*n* = 12): 0.05% tretinoin cream, on alternate nights + photoprotector	Histological and immunohistochemical: decreased corneal layer thickening of the dermis, decreased expression of p53 protein, increased type 1 collagen
	6 months	

Bravo, 2015^164^	Single group (*n* = 20): 20 mg 3 × /week	Clinical: improved skin quality in the opinion of the patient and researcher
	3 months	Histological: 60% increase in the thickness of collagen fibers in 65% of patients; improved elastic tissue fragmentation

#### Field cancerization – multiple actinic keratoses

The concept of field cancerization is old, and was based on histopathological studies of multifocal neoplasms of the oral mucosa that can coalesce, relapse, and develop new lesions. It was later extended to the skin, where UV radiation causes mutations in the p53 gene, resulting in multiple actinic keratoses and non-melanoma skin cancer.^164^, ^165^, ^166^, ^167^

Oral isotretinoin improves the clinical, histological, and immunohistochemical parameters of field cancerization, notably reducing the epidermal p53 protein.^162^, ^163^

The mechanism of action of retinoids in the prevention and treatment of non-melanoma skin cancer is not fully understood. They are known to have antiproliferative and anti-apoptotic actions, regulate keratinocyte differentiation and apoptosis, interfere with tumor initiation, reduce regulation of proto-oncogenes, and alter the expression of p53 and pro-apoptotic caspases.^168^, ^169^, ^170^ They work by preventing the proliferation of human papillomavirus (HPV), a known co-carcinogen.^171^

Studies involving oral retinoids have focused on the prevention and treatment of non-melanoma skin tumors that are only part of the cancerization process. Details of the studies that used it for treatment are presented in Table 5.^172^, ^173^, ^174^, ^175^

**Table 5:** Studies involving oral isotretinoin, non-melanoma skin cancer, actinic keratoses, and field cancerization

Author, year	Study design (*n*): indication	Dose/duration of treatment	Results
Haydey, 1980^173^	Case report (1): multiple keratoacanthomas	From 2–6 mg/kg/day, 16 weeks	No new lesions were observed
Peck, 1982^2^	Case series (3): multiple BCC	Mean of 1.5 mg/kg/day, 2.5–4 years	Regression of 9/65 lesions; no new lesions in 2–4 years
Levine, 1984^174^	Case report (1): SCC and multiple keratoacanthomas	2 mg/kg/day, 16 weeks	Regression of various lesions and reduction of new tumors
Peck, 1987^191^	Case reports (2): multiple BCC	2 mg/kg/day, 7 and 8 years	Regression of 15% of the lesions, decrease in the number of new lesions in the patient exposed to arsenic
Lippman, 1987^175^	Case series (4): keratoacanthoma and SCC		Patient 1 – total regression of the keratoacanthoma; 2 – partial regression; 3 – partial regression of subcutaneous mass; 4–70% reduction in SCC
Kraemer, 1988^182^	Case series (5): xeroderma pigmentosum	2 mg/kg/day, 2 years	121 tumors before treatment
			Reduction to 21 tumors in 2 years of treatment Suspension, follow-up for 1 year: 25 tumors
Peck, 1988^179^	Case series (12): BCC	Mean of 3.1 mg/kg/day, 8 months	Doses of 0.25–1.5 mg/kg/day were ineffective
Moshell, 1989^183^	Case series (5): xeroderma pigmentosum	2 mg/kg/day, 2 years	63% reduction in 25 tumors
			Suspension: 8 times more tumors
Lippman, 1992^187^	Series of cases (32/28): SCC	1 mg/kg/day + interferon alfa, 2 months	Partial response: 68%; total response: 25%
Tangrea, 1992^180^	Randomized, placebo-controlled clinical study (951): multiple SCC	10 mg/day, 3 years	No difference with placebo
Majewski, 1994^181^	Case series (4): multiple AK	10.4–0.5 mg/kg/day + calcitriol, 12 months	Patient 1: complete response; 2 and 3: 50–80% regression
Levine, 1997^190^	Randomized, placebo-controlled clinical study (525): 4 or more BCC or SCC	Retinol (25,000) × isotretinoin (5–10 mg/day) × placebo	No difference between drugs and placebo
Feldman, 2007^172^	Case report (1): multiple keratoacanthomas	40 mg/day, followed by acitretin and topical retinoid	Regression of some lesions
Troyanova, 2018^171^	Case report (1): epidermodysplasia verruciformis	0.33–1 mg/kg/day, 18 years	Reduction in the number of SCC
Ianhez, 2019^189^	Randomized clinical study (60): AK	10 mg/day × 0.05% tretinoin cream	Reduction in the number of AKs

SCC, squamous cell carcinoma; BCC, basal cell carcinoma; AK, actinic keratosis.

The reported indications for prevention include: multiple non-melanoma skin cancers (> 5 per year); multiple actinic keratoses (AKs); eruptive keratoacanthomas or occurring in transplanted and/or immunosuppressed patients, xeroderma pigmentosum, exposure to chronic phototherapy, and verruciform epidermodysplasia.^171^, ^176^, ^177^, ^178^, ^179^, ^180^, ^181^, ^182^, ^183^

Little is known about the use of retinoids in multiple AKs and field cancerization in immunocompetent or immunodepressed patients, at risk of developing non-melanoma skin cancer. The delimitation of field cancerization and the methodology to evaluate the effectiveness of therapies for its control is challenging. The most used method is treatment in a restricted, well-defined area, and counting of AKs with primary resolution. The recommended doses range from 0.25 to 6 mg/kg/day, lasting from months to years.^184^, ^185^

Considering that AKs are early signs of field cancerization and studies on oral retinoids are scarce,^186^, ^187^, ^188^ the present authors highlight the most recent study with oral isotretinoin, 10 mg/day *vs*. 0.05% cream tretinoin, on alternate nights. The results were similar, with a 28% decrease in the number of new AKs, after destruction of all visible AKs with cryotherapy. An improvement was observed in the immunohistochemical parameters with reduced expression of epidermal p53 and BAX proteins. The genes that encode these proteins undergo mutations induced by UV radiation, and start to act as tumor inducers instead of inducing apoptosis of keratinocytes, which were also mutated as a protective mechanism against carcinogenesis.^189^

Acitretin is mostly used in immunocompromised individuals, while isotretinoin is preferred for immunocompetent patients and women with the potential to become pregnant, due to its shorter half-life. Low doses are less effective to justify its use in the treatment of non-melanoma skin cancer. However, for prevention in high-risk patients, high doses and long-term treatment should be discouraged, due to the risk of adverse events; low doses are justified to stabilize field cancerization.^190^, ^191^, ^192^

#### Hair and scalp diseases

##### Frontal fibrosing alopecia (FFA)

FFA is characterized by the retreat of the line of hair implantation and loss of eyebrows and, at times, body hair, and also by facial papules, red glabellar spots, depression of the frontal veins, and association with lichen planus pigmentosus.^193^, ^194^, ^195^, ^196^ It is an epidemic, since in two decades it is no more a “recently described” disease and has become the most common scarring alopecia, according to a multicenter study.^197^

A retrospective study compared isotretinoin 20 mg/day (*n* = 29), acitretin 20 mg/day (*n* = 11), and finasteride 5 mg/day (*n* = 14), for an average of 13.5 months. The objectives of not increasing the distance between the glabella and the hair line after 12 months and maintaining the results after one year of treatment were achieved in 76% and 73% *vs*. 72% and 73% of patients treated with isotretinoin and acitretin, respectively, and in 43% of those treated with finasteride.^198^ Another retrospective study included 291 patients with lichen planus pilar, of whom 26 had FFA. Of these, seven were treated with isotretinoin, 20 mg/day and four with isotretinoin associated with finasteride or dutasteride. Six had a complete response with isotretinoin, as well as the four who received the combined treatment. All patients used topical tacrolimus and clobetasol concomitantly. The response was assessed by clinical photos, perifollicular scaling, and papules, without any objective method.^199^ In three patients treated with isotretinoin 20 mg/day in the first month and 0.5 mg/kg/day in the second and third months, the facial papules improved and regressed after 15 days. However, signs of disease activity, erythema, and perifollicular scaling remained.^200^ A later study reported reduction of facial papules after two to four months with isotretinoin, 10 mg every other day, in ten patients.^201^ Recently, two cases treated with isotretinoin, 10 mg/day, presented improvement in the papules after 30–45 days of treatment.^202^ In the last three studies, the progression of FFA was not evaluated. To date, data in the literature do not allow an absolute conclusion about the efficacy of this drug in FFA. Further studies are needed.

##### Dissecting cellulitis (DC)

DC is a neutrophilic primary scarring alopecia with follicular pustules, nodules, intercommunicating abscesses, and irreversible follicular destruction. It can constitute the tetrad of follicular occlusion when associated with pilonidal cyst, hidradenitis, and acne conglobata.^203^, ^204^

The first report of therapeutic success with isotretinoin, at a dose of 0.5 mg/kg/day for three months, showed relapse and the need for two more cycles of 1 mg/kg/day until remission.^205^ Three patients with DC received 1 mg/kg/day, then 0.75 mg/kg/day, for maintenance, for nine to 11 months, without recurrence after ten months (one patient) and after two years and six months (two patients). The authors suggested high doses and prolonged treatment to reduce relapses.^206^ A retrospective study, including seven patients treated with a dose of 0.75 mg/kg/day for nine to 12 months, found no recurrence at 16–42 months.^207^ A retrospective study of 51 patients treated with 0.5–0.8 mg/kg/day observed complete remission in 92% of the patients after three months and frequent relapses.^208^ In another report of 28 patients treated with a mean dose of 30 mg/day, seven had reduced inflammatory activity; relapse and need for retreatment were not specified.^208^

Doses of 10 mg/day to 1 mg/kg/day, duration, maintenance doses, and varying associations have been reported in the literature. Despite the small number of reports, frequent relapses, and few studies with long follow-up, a SR concluded that, even without evidence, oral isotretinoin is considered the treatment of choice for DC.^204^

##### Quinquaud folliculitis decalvans (QFD)

Quinquaud folliculitis decalvans is a rare, chronic, and recurrent neutrophilic scarring alopecia that affects young adults of both sexes. It is characterized by fibrotic plaques of alopecia with tufts of hair on the periphery, erythema, follicular pustules, flaking, and crusts. Its etiology is unclear, and no therapy is capable of inducing prolonged remission. Isotretinoin can act by inhibiting the migration of neutrophils and modulating innate immunity against Gram-positive bacteria, through negative regulation of TLR-2. Isotretinoin is widely cited, with differences regarding efficacy, safety, time to remission, and relapses.^209^

In a retrospective study involving 82 patients, 16 (20%) used isotretinoin; eight (50%) improved, but the duration of the response was only three months.^210^ A multicenter, prospective study included 60 patients with QFD and different treatments; 15 (25%) were treated with isotretinoin for three months, with no difference in efficacy compared with the combination of rifampicin and clindamycin in the five-year follow-up. The authors developed a therapeutic protocol for QFD, suggesting isotretinoin only for severe cases, when a response is not maintained with other treatments.^211^ Another study assessed 39 patients treated with isotretinoin 0.1–1.02 mg/kg/day for a mean of 2.5 months; 82% presented a partial or complete response. Doses above 0.4 mg/kg/day and lasting more than three months have been associated with the best response.^212^

Recent SRs have shown controversial results. One concluded that isotretinoin is the treatment with the largest number of publications, despite the limited response^213^; the other concluded that the ideal option is the combination of clindamycin and rifampicin, with level of evidence 3.^214^

### Other diseases with keratinization and inflammation disorders

The modulation of the inflammatory response and keratinocyte hyperproliferation and differentiation justifies the indication of isotretinoin for keratinization disorders with inflammation and difficult treatment. There are case reports and citations in reviews, with no conclusions about dose and duration. Genodermatoses need continuous treatment and there are no data on long-term risks.

## Results of studies justifying recommendation

### Pityriasis rubra pilaris

A chronic, papular-desquamative disease, of unknown familial or acquired etiology. Its treatment is difficult, and includes UVB associated with coal tar, topical corticosteroids, calcipotriene, keratolytics, oral retinoids, methotrexate, azathioprine, and cyclosporine. The use of isotretinoin has been reported since the 1980s, with good results.^215^, ^216^ A recent SR included 182 studies and 475 patients. Among those treated with retinoids, isotretinoin led to a good response in 61%; etretinate in 47%; and acitretin in 24%. The authors suggested that the first-line treatment is isotretinoin, followed by methotrexate and immunobiologicals. Cutaneous xerosis is aggravated by the drug and requires the use of emollients.^217^

### Cutaneous lupus erythematosus (LE)

Isotretinoin 0.2–1 mg/kg/day was indicated as an option for refractory cases of subacute LE, chronic LE, and hyperkeratotic forms, with efficacy similar to hydroxychloroquine. However, adverse events and faster relapse are more frequent; in practice, it is little used. Contraindicated in the association of LE and Sjogren's syndrome.^218^

### Generalized granuloma annular

A non-infectious granulomatous disease, with papules and plaques, of unknown cause. There is no effective treatment. There are reports in the literature of the use of isotretinoin 0.5 mg/kg/day for two to six months, in the case of generalized forms, with good response but with recurrence; maintenance at a low daily dose is suggested.^219^

### Human papilloma virus (HPV)/condyloma acuminatum

Oral isotretinoin 0.5–1 mg/kg/day was effective for condyloma of the cervix and mucocutaneous warts, especially when flat and recalcitrant.^220^, ^221^ There may be no response, even at a dose of 1 mg/kg/day; however, it contributes to the reduction of the volume or multiplicity of lesions, favoring supporting treatments.

### Darier's disease

A genetic dermatosis, with extensive areas of hyperkeratotic papules and plaques. Case reports and a review article reported improvement with isotretinoin at daily doses of 0.2–0.7 mg/kg. As a chronic disease, there is a need for continuous use with surveillance of hepatotoxicity, hypertriglyceridemia, and teratogenicity.^222^, ^223^, ^224^

### Others

Varied and off-label indications, with no possibility of conclusions on efficacy and safety as they are single reports, include: aquagenic keratoderma, oral mucosa ulcer perioral dermatitis, Galli-Galli disease, acne-like rash secondary to vemurafenib, dermatophytosis, lichen planopilaris and lichen planus pigmentosus, Cushing's disease, sebaceous hyperplasia, Fordyce granules, multiple steatocystoma, reticulated confluent papillomatosis (Gougerot-Carteaud), and erosive pustular dermatosis of the scalp.^225^, ^226^, ^227^, ^228^, ^229^, ^230^, ^231^, ^232^, ^233^, ^234^, ^235^, ^236^, ^237^, ^238^, ^239^

Table 6 presents a summary of approved and off-label indications for oral isotretinoin in dermatology, regarding doses and treatment times, reported in clinical studies, guidelines for conduct, and consensuses.

**Table 6:** Summary of doses and treatment time for approved and unapproved indications for oral isotretinoin, according to clinical studies, case series, case reports, and consensuses

Indications	Oral isotretinoin dose	Treatment time
Acnes grades III and IV or unresponsive to previous treatments^1^, ^2^, ^4^, ^5^, ^36^, ^40^, ^41^, ^42^, ^43^, ^46^, ^51^, ^52^, ^53^, ^54^, ^56^, ^58^, ^59^, ^64^, ^65^, ^67^, ^71^, ^72^, ^73^, ^74^, ^79^, ^80^, ^83^	0.5–1 mg/kg/day	Up to a dose of 120–150 mg/kg/day or until complete regression of the lesions
Acne – “low daily dose”^10^, ^38^, ^48^, ^55^, ^61^, ^62^, ^69^, ^71^, ^80^, ^81^, ^82^	0.1–0.5 mg/kg/day	Up to 18 months or up to 1 to 2 months after lesion resolution
Acne flares^100^, ^101^	Low dose (up to 8 weeks) associated with prednisone	8 weeks low dose oral isotretinoin
		Prednisone: 0.5–1 mg/kg/day for 2 weeks or until resolution of the flare
Severe and extensive acne associated with macrocomedones^36^, ^42^, ^100^, ^101^	01–0.2 mg/kg, 8 weeks, may or may not be increased gradually with fractionated prednisone used in the first 2 to 4 weeks	Low dose: 8 weeks
		Prednisone: 2–4 weeks
Rosacea^112^, ^113^, ^114^, ^115^, ^116^, ^117^, ^118^, ^119^	0.25 to 0.3 mg/kg	4 months
	Selected cases, “microdoses”: mean 0.07 mg/kg/day, up to 33 months	Microdose: up to 33 months
Rosacea fulminans^120^	0.2–0.5 mg/kg, increased up to 0.5–1 mg/kg, associated with prednisone 40–60 mg/day	3–4 months
Phymatous rosacea^121^	20 mg/day active phase, maintenance at 10 mg/day	6 months for active phase
Ocular rosacea^125^, ^126^	10 mg/day	4 months
Seborrheic dermatitis^139^, ^140^, ^141^, ^142^, ^143^, ^144^	0.05–0.51 mg/kg/day	Mean of 33 weeks
Pustular psoriasis^148^, ^149^, ^150^	Children: 0.75 mg/kg/day	4 months
	Adults: 1.5–2.0 mg/kg/day	
Photoaging^159^, ^160^, ^161^, ^162^, ^163^, ^164^	10–20 mg 3 × /week	2–6 months
Field cancerization^190^	10 mg/day	6 months
Frontal fibrosing alopecia^205^, ^206^	20 mg/day	Mean: 13 months
Facial papules of frontal fibrosing alopecia^207^, ^208^, ^209^	10 mg every other day or 20 mg/day	2–4 months
Dissecting cellulitis^212^, ^213^, ^214^, ^215^, ^216^, ^217^, ^218^, ^219^, ^220^, ^221^	0.5–1 mg/kg/day	3–12 months
	Low dose – 10 mg/day	
Pityriasis rubra pilaris^222^, ^223^, ^224^	0.5–2 mg/kg/day	16–24 weeks
Cutaneous lupus erythematosus^225^	0.2–1 mg/kg/day	5 months
Generalized granuloma annulare^226^	0.5 mg/kg/day	2–6 months, maintenance at lower doses
Condyloma acuminatum and recalcitrant warts^227^, ^228^	0.5 mg/kg/day (cervical condyloma)^227^	12 weeks
	0.5–1 mg/kg/day^228^	Mean: 3 months
Darier's disease^229^, ^230^	0.2 mg/kg at the beginning, increase to 0.5–1 mg/kg, according to the hue of the lesion	Continuous use (genodermatosis)

### Perspectives

For the cure of acne, the present authors consider it relevant to expand the prescription of isotretinoin for adolescents and adults, as well as the prescription of anti-androgens (contraceptives and spironolactone) for adult women, thus reducing the prescription of oral antibiotics, considering the growing alert about bacterial resistance. The still very high use of these drugs is worrisome, lasting from over six months up to one year or more (mean: 331 days), according to a 2016 study that highlights the lack of knowledge of or disregard of the recommendations on the rational use of antibiotics.^240^ It is known that there is no minimum age to prescribe isotretinoin, since acitretin is indicated for children of any age to treat severe keratinization disorders. However, it is necessary to guide the patient and family that use in pre-adolescents may imply the need for new treatment cycles; a new cycle can begin after three months.^241^ Until now, and considering that the patent of isotretinoin has expired, there appears to be no interest from the pharmaceutical industry in conducting multicenter, randomized and controlled studies aimed at future approvals for other dermatoses. Only studies with appropriately-sized samples and high quality methodology will allow approval by regulatory agencies and the possibility of establishing levels of evidence in accordance with international standards.^242^, ^243^, ^244^, ^245^ Perhaps the development of a new oral retinoid that can meet other indications, in addition to acne and psoriasis, could expand the use of these drugs in dermatology.

## Conclusions

This consensus aims to guide dermatologists on the use of oral isotretinoin for the benefit of patients. There is only level I evidence (SR and meta-analysis) with respect to efficacy and safety, ensured by adverse event monitoring, in the treatment of acne vulgaris. For rosacea, its use in low daily doses is mentioned in one SR, without mentioning the level of evidence. For the other indications, the literature is scarce, generally based on case reports, some even anecdotal, and rare randomized clinical studies with small samples (seborrheic dermatitis, photoaging), with no possibility of determining the level of evidence. However, some dermatological conditions that are difficult to control and for which oral isotretinoin was attempted due to its multiple mechanisms of action are worth mentioning. There was a 100% consensus among the authors of this manuscript that off-label indications are expanding and should be included. In turn, in the opinion of the authors, indications for purely esthetic purposes or oil control are not recommended, particularly for women of childbearing age. Finally, common sense is needed to prescribe a teratogenic drug, particularly for off-label prescriptions, in which the responsibility lies entirely with the physician.

## Financial support

Brazilian Society of Dermatology – SBD.

## Authors’ contributions

Ediléia Bagatin: Approval of the final version of the manuscript; design and planning of the study; drafting and editing of the manuscript; critical review of the literature; critical review of the manuscript.

Caroline Sousa Costa: Approval of the final version of the manuscript; critical review of the literature; critical review of the manuscript.

Marco Alexandre Dias da Rocha: Approval of the final version of the manuscript; critical review of the literature; critical review of the manuscript.

Fabíola Rosa Picosse: Approval of the final version of the manuscript; critical review of the literature; critical review of the manuscript.

Cristhine Souza Leão Kamamoto: Approval of the final version of the manuscript; drafting and editing of the manuscript; critical review of the literature; critical review of the manuscript.

Rodrigo Pirmez: Approval of the final version of the manuscript; drafting and editing of the manuscript; critical review of the literature; critical review of the manuscript.

Mayra Ianhez: Approval of the final version of the manuscript; drafting and editing of the manuscript; critical review of the literature; critical review of the manuscript.

Hélio Amante Miot: Approval of the final version of the manuscript; design and planning of the study; drafting and editing of the manuscript; critical review of the literature; critical review of the manuscript.

## Conflicts of interest

None declared.
